# A Crucial Role for Kupffer Cell-Derived Galectin-9 in Regulation of T Cell Immunity in Hepatitis C Infection

**DOI:** 10.1371/journal.pone.0009504

**Published:** 2010-03-04

**Authors:** John A. Mengshol, Lucy Golden-Mason, Tomohiro Arikawa, Maxwell Smith, Toshiro Niki, Ryan McWilliams, Jessica A. Randall, Rachel McMahan, Michael A. Zimmerman, Manu Rangachari, Evgenia Dobrinskikh, Pierre Busson, Stephen J. Polyak, Mitsuomi Hirashima, Hugo R. Rosen

**Affiliations:** 1 Department of Medicine, Division of Gastroenterology & Hepatology, University of Colorado School of Medicine, Aurora, Colorado, United States of America; 2 Denver Veterans Affairs Medical Center, Denver, Colorado, United States of America; 3 Division of Clinical Immunology, University of Colorado School of Medicine and National Jewish Hospital, Denver, Colorado, United States of America; 4 Department of Pathology, University of Colorado School of Medicine, Denver, Colorado, United States of America; 5 Department of Immunology and Immunopathology, Kagawa Medical University, Kagawa, Japan; 6 GalPharma, Kagawa, Japan; 7 Division of Transplant Surgery, University of Colorado School of Medicine, Aurora, Colorado, United States of America; 8 Center for Neurologic Diseases, Brigham and Women's Hospital, Harvard Medical School, Boston, Massachusetts, United States of America; 9 Institut de Cancérologie Gustave Roussy, Villejuif, France; 10 Division of Laboratory Medicine, University of Washington, Seattle, Washington, United States of America; New York University, United States of America

## Abstract

Approximately 200 million people throughout the world are infected with hepatitis C virus (HCV). One of the most striking features of HCV infection is its high propensity to establish persistence (∼70–80%) and progressive liver injury. Galectins are evolutionarily conserved glycan-binding proteins with diverse roles in innate and adaptive immune responses. Here, we demonstrate that galectin-9, the natural ligand for the T cell immunoglobulin domain and mucin domain protein 3 (Tim-3), circulates at very high levels in the serum and its hepatic expression (particularly on Kupffer cells) is significantly increased in patients with chronic HCV as compared to normal controls. Galectin-9 production from monocytes and macrophages is induced by IFN-γ, which has been shown to be elevated in chronic HCV infection. In turn, galectin-9 induces pro-inflammatory cytokines in liver-derived and peripheral mononuclear cells; galectin-9 also induces anti-inflammatory cytokines from peripheral but not hepatic mononuclear cells. Galectin-9 results in expansion of CD4^+^CD25^+^FoxP3^+^CD127^low^ regulatory T cells, contraction of CD4^+^ effector T cells, and apoptosis of HCV-specific CTLs. In conclusion, galectin-9 production by Kupffer cells links the innate and adaptive immune response, providing a potential novel immunotherapeutic target in this common viral infection.

## Introduction

Approximately 200 million people throughout the world are infected with hepatitis C virus (HCV) [1]. HCV infection establishes chronicity in the vast majority of patients [2], and is a leading etiology of cirrhosis, liver cancer and indication for liver transplantation [3]. Although new therapies have improved the rates of sustained response, a large proportion of patients still fail to respond to antiviral treatment or develop significant drug toxicity [4], thus remaining at risk for disease progression. Enhanced understanding of HCV-host interactions is required to combat this virus and to develop improved therapies.

Galectins are evolutionarily conserved glycan-binding proteins with diverse roles in innate and adaptive immune responses [5]. Galectins demonstrate a shared structural fold and at least one conserved carbohydrate-recognition domain (CRD) of about 130 amino acids that recognizes glycans and mediates carbohydrate binding. Moreover, at least 15 galectins have been identified in mammals [6] http://www.nature.com/ni/journal/v9/n6/full/ni.f.203.html - B8#B8. “Prototype” galectins have CRDs that can dimerize, whereas so-called “tandem-repeat galectins” contain two distinct CRDs in tandem in a single polypeptide chain, separated by a linker of up to 70 amino acids. Accumulating evidence indicates a critical role of the tandem repeat-type galectin-9 in regulating the fate of effector T cells, specifically by binding to the T cell immunoglobulin domain and mucin domain protein 3 (Tim-3) to induce apoptosis of some T cell subsets [5] [7], as well as promoting the differentiation of regulatory T cells (Treg) expressing FoxP3 [8]. Recently we found that Tim-3 was increased on exhausted T cells in chronic hepatitis C infection and may provide a novel therapeutic target [9].

As the natural ligand for Tim-3, galectin-9 is widely distributed throughout various tissues, being particularly abundant in the liver [10]. The liver is among the most interesting and potent immunologic organs, rivaled only by what are considered the true immune organs, such as thymus and lymph nodes [11]. The liver is exposed continuously to a diverse and large antigenic load, including pathogens, toxins, and tumor cells, as well as dietary and commensal proteins. The liver must be actively immunocompetent and simultaneously control inappropriate inflammatory responses to harmless antigens encountered in the portal circulation, thus being able to selectively induce immunity or tolerance to antigens [12]. Moreover, numerous studies have identified the liver as a major site for apoptosis and removal of peripheral CD8^+^ T cells undergoing antigen induced cell death (AICD) [13].

We speculated that the dysfunctional antiviral T cell responses characteristic of HCV infection–expansion of suppressive Tregs [14], [15] and impaired HCV-specific cytotoxic T lymphocytes (CTL) [16] [17] [9], particularly within the intrahepatic compartment–might be related to increased expression of galectin-9. In the current study, we determined the circulating and hepatic expression of galectin-9 in chronic HCV infection, finding that Kupffer cells (KC) are the predominant source of galectin-9 within the liver, and that its expression is upregulated in HCV-infected livers relative to normal livers. Furthermore, we find that macrophage-derived galectin-9 production is induced by IFN-γ, and that galectin-9, in turn, leads to the production of pro-inflammatory cytokines. In addition, galectin-9 induces the expansion of regulatory T cells in chronic HCV infection in a TGF-β-dependent manner, as well as apoptosis of HCV-specific CTLs via activation of caspase 8. We propose a novel paradigm whereby Kupffer cell-derived galectin-9 regulates T cell responses within the liver, providing novel insights into the bridge between innate and adaptive immunity, mechanisms of viral persistence, and potential immunotherapeutic targets for HCV infection. Moreover, given the broad array of responses induced by galectin-9, including potentially detrimental processes, this pathway may be important in mediating immunopathology in other hepatic diseases.

## Results

### Circulating Galectin-9 Levels Elevated in Chronic HCV Patients

**Figure 1:**
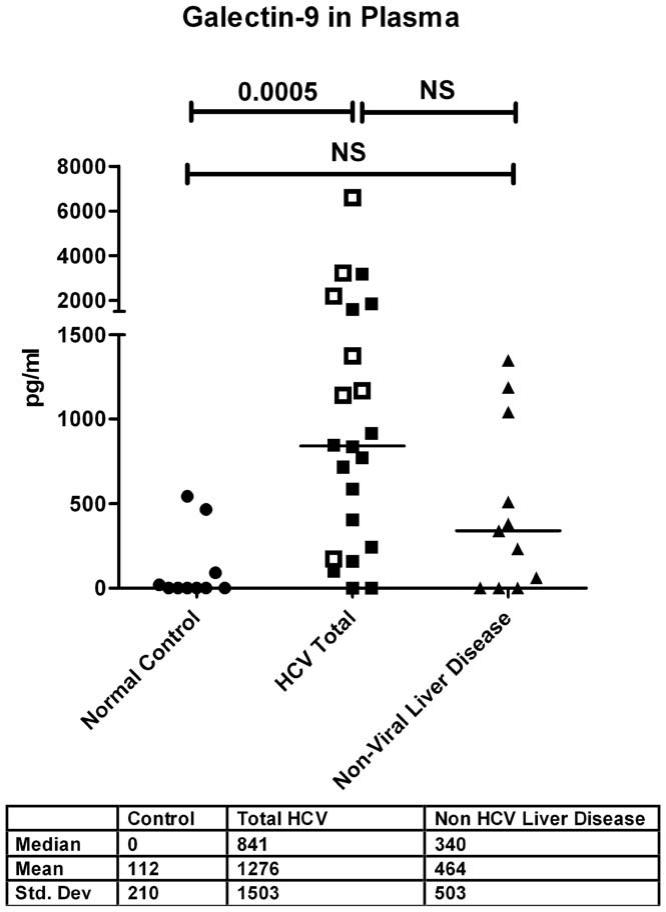
Galectin-9 is elevated in the plasma of patients with chronic HCV and higher levels are seen in hepatocellular carcinoma (HCC). Galectin-9 levels were analyzed in the plasma of 10 normal controls, 22 patients with HCV and 11 patients with non-viral causes of liver disease using a sandwich ELISA [50]. Seven patients with HCV and HCC are denoted by open squares; they had significantly higher galectin-9 levels compared to HCV patients without HCC, p = 0.0334. Plasma from 11 patients with non-viral liver disease was analyzed. Three patients had alcoholic liver disease, 3 patients had Primary Biliary Cirrhosis, 3 patients had autoimmune hepatitis and 2 patients had non-alcoholic steatohepatitis. P- values were calculated using the two tailed Mann-Whitney test. NS denotes non-significant, p values>0.05.

Given the recent demonstration that expression of negative immune regulator Tim-3 is increased on exhausted T cells in patients with HCV [9] and that galectin-9 is a ligand for Tim-3 [7], we analyzed the circulating levels of galectin-9 in the plasma from patients with chronic HCV, non-viral liver disease and normal controls. We found that galectin-9 levels were strikingly elevated in HCV patients (median 841 pg/ml in HCV vs. 0 in normal controls, p = 0.0005, **Figure 1**). Importantly, HCV-positive patients with all stages of fibrosis demonstrated elevated levels of circulating galectin-9 (data not shown). Moreover, HCV-infected patients with hepatocellular carcinoma had even higher levels when compared to HCV alone (n = 7, median 1376 vs. n = 15 median 715 pg/ml; p = 0.0288). Levels in 11 patients with non-viral liver disease were elevated (median 340 pg/ml) but to lower levels than HCV patients and were not significantly different from normal patients (p = 0.06) or HCV patients.

### Galectin-9 Expression Up-Regulated on Kupffer Cells in Livers of HCV-Infected Patients

**Figure 2:**
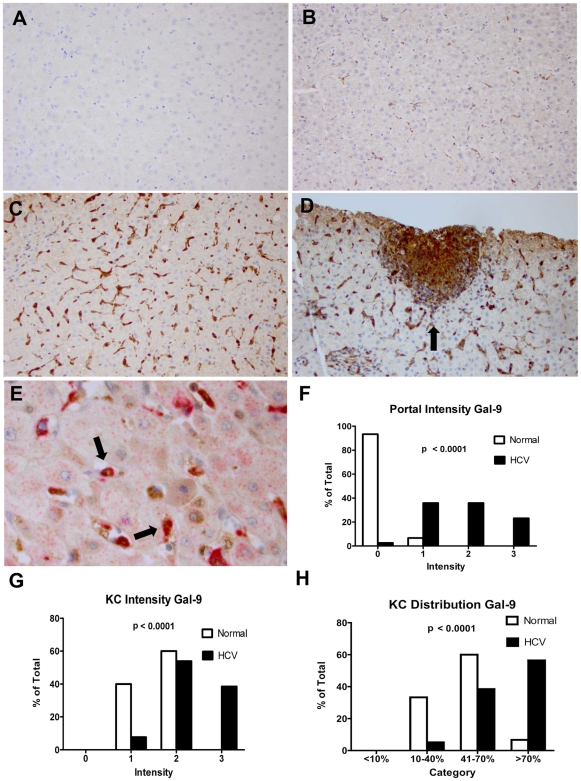
HCV patients have increased Galectin-9 in Kupffer cells and periportal areas. Immunohistochemistry of paraffin embedded samples (see Methods for details) was used to analyze 39 samples from HCV-infected patients and 15 normal subjects for galectin-9 staining. Staining for CD68 was used to identify Kupffer cells. The analyzing pathologist (M.S.) was blinded to patient identity and medical history. (**A**) Negative control with secondary antibody only, 20× magnification. (**B**) Galectin-9 staining in normal liver (brown) 20×; (**C**) Galectin-9 staining in a HCV patient 20×, (**D**) Periportal galectin-9 staining in HCV patient, 200×. (**E**) Co-localization of galectin-9 (brown) and CD68 (red) 600×. (**F–H**) Intensity of galectin-9 was scored from 0 to 3, and frequency was scored 0 (<10%), 1 (10–40%), 2 (40–70%), 3 (>70%). Distribution of scores by category expressed as a percent of the total patients comparing normal patients to patients with HCV. P-values calculated by the Chi-square test for trend.

In order to determine the cell types producing galectin-9 in chronic HCV, we analyzed paraffin-embedded liver biopsy and liver resection specimens for galectin-9 protein by immunohistochemistry (IHC). Using CD68 as a marker for KC, we found that KC had the highest staining for galectin-9 (**Figure 2a–e**). KC constitute a cellular component of the hepatic sinusoids, anchored to the luminal site of the endothelium and, thus, exposed to the bloodstream [18]. HCV patients had higher intensity and frequency of KC staining compared to normal controls (**Figure 2g and h**, p<0.0001 for both). The majority of KC are located in periportal regions where they have greater phagocytic activity and are larger than those found in the perilobular region [18], resulting in a zonal distribution with differential KC function. We observed that 93% of normal control livers demonstrated no galectin-9 staining in the periportal regions, whereas virtually all the patients with HCV infection, regardless of grade of inflammation or stage of fibrosis, stained positively (**Figure 2f**). To confirm our staining, we also performed immunofluorescence and confocal microscopy on paraffin-embedded liver biopsy sections from HCV patients using antibodies to galectin-9, albumin and CD68 **(Figure S1)**. We did not see staining of galectin-9 in hepatocytes (albumin positive) **(Figure S1A)**; we again saw co-staining of CD68 and galectin-9, confirming that KC are galectin-9 positive **(Figure S1B)**.

### IFN-γ Induces Galectin-9 Production by Human Monocytes and Macrophages

Next, based on the IHC data, we determined which factors might stimulate production of galectin-9 by monocytes/macrophages. Prior work has indicated that galectin-9 expression can be induced in various cells such as endothelial cells, fibroblasts and astrocytes [19] [20] [21] by IFN-γ or IL-1β. There are three isoforms of galectin-9 according to the size of the linker peptide connecting two CRDs [22]: long (Gal-9L, MW 39.5 kD), medium (Gal-9M, 35.9 kD), and short (Gal-9S, 34.7 kD) isoforms. Although these isoforms have shown comparable eosinophil chemo-attractant activity [23], little is known about their differences in other biological functions. Specifically, among the three isoforms, the medium and long gal proteins were upregulated by IFN- γ in human fibroblasts [21].

**Figure 3:**
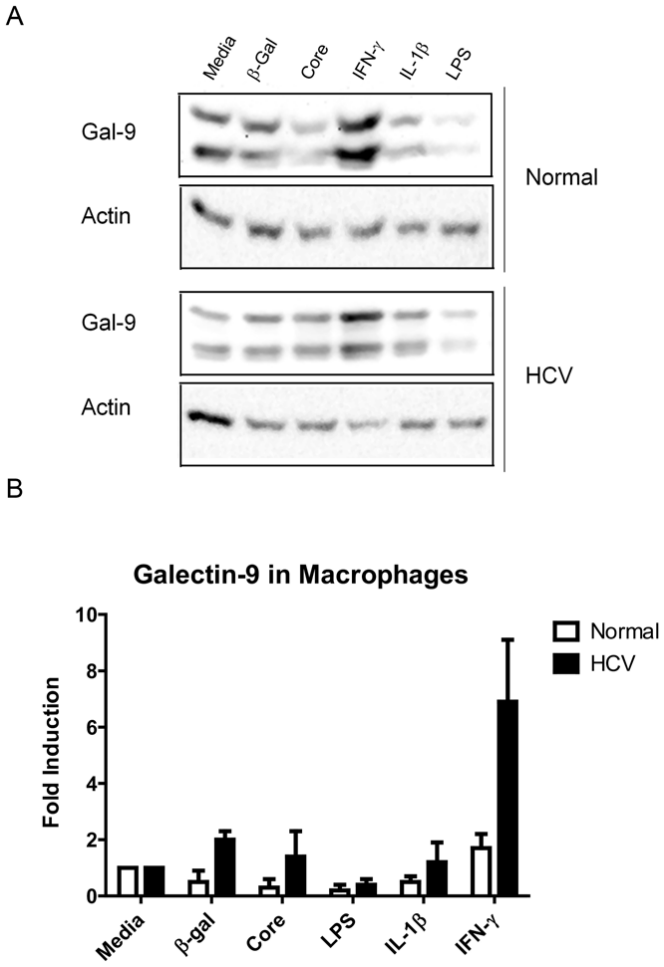
Interferon gamma increases galectin-9 levels in macrophages. CD14 positive cells from five normal controls and five HCV patients were bead selected from PBMCs and cultured for 48 hours with 25 ng/ml of MCSF to differentiate them into macrophages. Cells were stimulated for 48 hours with the stimuli shown at 2×10^6^/ml. IFN-γ was used at 25 ng/ml, HCV core and β-gal were used at 10 ug/ml, LPS was used at 100 ng/ml, and IL-1β was used at 10 ng/ml. Cell lysates were prepared and analyzed by Western Blot. (**A**) Western blot from one representative normal patient and one representative HCV patient to galectin-9 and β-Actin. (**B**) Fold induction was determined by densitometry using β-Actin as a loading control (n = 4 normal, and 3 HCV-positive patients).

We found that peripherally derived macrophages differentiated with mCSF produced a basal level of galectin-9 and that IFN-γ was a strong inducer of galectin-9 at 48 hours (**Figure 3a**). The medium and long isoforms of galectin-9 were induced preferentially. The fold induction of galectin-9 was higher in patients with chronic hepatitis C (7 fold) compared to normal controls (1.8 fold). Production of galectin-9 from macrophages was not significantly stimulated by IL-1β, LPS or HCV core protein.

### Galectin-9 Induces Pro-Inflammatory Cytokines in Liver-Derived and Peripheral Mononuclear Cells

**Figure 4:**
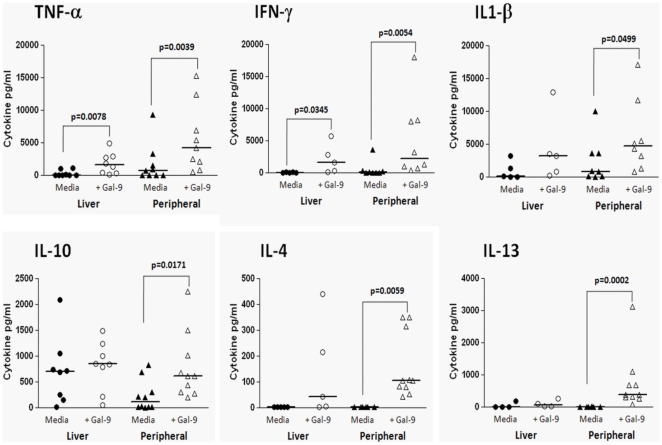
Galectin-9 induces cytokine secretion from liver-derived and peripheral mononuclear cells. Hepatic (n = 8) or peripheral (n = 8) mononuclear cells (2×10^6^/ml) were cultured in 96-well plates (200 ul/well) for 48 hours in media alone (RPMI+10% human serum) ± galectin-9 (5 ug/ml). After the culture period supernatants were collected and cytokine levels were measured using cytokine multiplex (Luminex™) technology as described in materials and methods. P-values were calculated using the Wilcoxan matched-pairs signed rank test.

Having demonstrated that HCV-infected patients have higher circulating and intrahepatic levels of galectin-9, we next explored the effects of recombinant galectin-9 on whole peripheral blood mononuclear cells (PBMCs) and liver-derived mononuclear cells from normal healthy subjects and HCV-infected patients. As shown in **Figure 4**, 48 hours of galectin-9 treatment induced an array of pro-inflammatory mediators, such as TNF-α, IL-1β, and IFN-γ from peripheral and liver derived mononuclear cells of study subjects. Intracellular cytokine staining demonstrated that TNF-α was produced by CD14+ monocytes, T and NK cells, but not B cells following stimulation with galectin-9 (data not shown). Moreover, galectin-9-induced cytokine production was not due to LPS contamination and was TLR4 independent, since a TLR4 blocking antibody had no effect on cytokine production (data not shown).

Production of the anti-inflammatory cytokines IL-4, IL-10, and IL-13 was induced in galectin-9-treated PBMCs but not hepatic mononuclear cells (**Figure 4** **)**. Liver-derived mononuclear cells produced higher basal levels of IL-10 than PBMC, consistent with prior studies showing more IL-10 production from KC [24]–[25]. Taken together, these data implicate galectin-9 as a key regulator of hepatic immunity by preferentially inducing pro-inflammatory cytokines instead of hepatoprotective factors such as IL-4 and IL-13 in the liver.

### Galectin-9 Expands Regulatory T Cells (Tregs)

CD4^+^ CD25^+^ Foxp3^+^ regulatory T cells are crucial for negatively regulating immune responses, and their immunosuppressive effects are mediated by both direct cell-to-cell contact and secretion of anti-inflammatory cytokines [26]. There is ample evidence that Treg cells are increased in frequency in HCV infection [27] [14]; however, the precise mechanism by which this occurs is unclear. In other model systems, it has been shown that blocking Tim-3 results in a significant reduction of Treg suppressive activity in vitro [28], and Galectin-9 up-regulates both Foxp3 mRNA expression and Treg differentiation induced by TGF-β1 [8]. Administration of galectin-9 improves two model autoimmune conditions, experimental allergic encephalitis and collagen-induced arthritis [7] [8]. Moreover, galectin-9-deficient mice demonstrate significantly decreased Tregs, indicating that Galectin-9, in part, regulates differentiation, maintenance or expansion of Tregs expressing Foxp3 [8].

**Figure 5:**
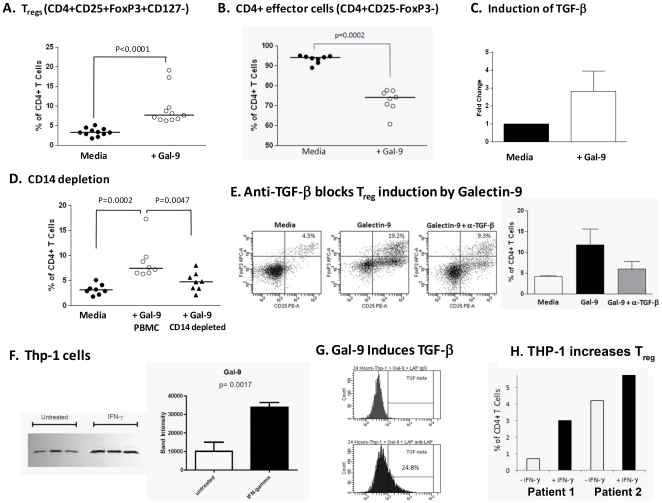
Galectin-9 induces regulatory T cells (T_regs_) via TGF-β. Peripheral mononuclear cells (2×10^6^/ml) isolated from 5 chronic HCV patients and 6 normal controls were cultured for 5 days in media alone (RPMI+10% human serum) ± galectin-9 (5 ug/ml). After the culture period Treg levels were estimated using flow cytometry. Tregs were defined as CD4^+^CD25^+^FoxP3^+^CD127^−^ T cells. Culture in the presence of galectin-9 induced Tregs (**A**) and a concomitant reduction in the CD4^+^CD25^−^FoxP3^−^ effector population was observed (**B**). (**C**) CD14^+^ monocytes were isolated from 3 chronic HCV patients and cultured in for 48 hours in media alone (RPMI+10% human serum) ± Galectin-9 (5 ug/ml). Galectin-9 increased the relative expression of TGF-β1 mRNA as analyzed by real time PCR. (**D**) Depletion of CD14^+^ cells from PBMC (8 normal patients) cultures treated with Galectin-9 attenuated the induction of Tregs at 5 days. (**E**) The addition of anti-TGF-β antibody at day 0 and day 2 of culture with PBMC and galectin-9 blocked induction of the Treg population. Representative flow plots are shown; the bar graphs are derived from three separate experiments. (**F**) THP-1 monocyte cell line constitutively expresses Galectin-9 which is upregulated by IFN-γ (25 ng/ml for 48 hours). Both the western blot and the densitometry analysis are shown, samples analyzed in triplicate. (**G**) Galectin-9 treatment induces TGF-β production from THP-1 cells. THP-1 cells were cultured with galectin-9 at 2.5 ug/ml for 24 hours then incubated with the latency associated peptide (LAP) then anti-LAP-PE to detect TGF-β. (**H**) IFN-γ treated THP-1 cells induce Treg from CD4^+^ T cells. Cells were cultured at a 1∶1 ratio for 5 days. Tregs were defined as CD4^+^CD25^+^FoxP3^+^CD127^−^ T cells and graphed as a % of total CD4^+^ cells. P-values were calculated using the Wilcoxan matched-pairs signed rank test.

To elucidate a role for galectin-9 in chronic HCV, we stimulated whole PBMCs from patients with chronic infection and normal controls, and the percentage of CD4^+^CD25^+^FoxP3^+^ CD127^low^ regulatory T cells was compared after 5 days in culture. PBMCs used in these experiments were not separated into subgroups in order to reflect physiological conditions most closely. Prior work has shown that low levels of CD127 expression can identify more than 95% of FoxP3^+^ T cells that have highly immunosuppressive activity [29]. Galectin-9 consistently expanded CD4^+^CD25^+^FoxP3^+^CD127^low^ Tregs in both normal and HCV-infected patients (**Figure 5a**). In keeping with data indicating that Treg cells can suppress the proliferation of effector T cells [30], we found that CD4^+^CD25^−^ effector cells contracted in culture with galectin-9 (**Figure 5b**).

Because it has been shown that TGF-β1 can convert CD4^+^CD25^−^ naïve T cells into Tregs *in vitro* [31], we explored whether it mediated the galectin-9 effect. Galectin-9 increased the relative expression of TGF-β1 mRNA in CD14+ monocytes (**Figure 5c**), and CD14-depleted PBMCs treated with galectin-9 showed decreased expansion of Tregs (**Figure 5d**). Moreover, blockade of the 5 day whole PBMC cultures by anti-TGF–β attenuated the ability of galectin-9 to expand Tregs (**Figure 5e**).

Because THP-1 cells show properties of human monocyte-derived macrophages, we characterized their expression of galectin-9 protein and mRNA which we found to be constitutive, as well as induced by IFN-γ **(** **Figure 5f** **, Figure S2)**. Additionally, galectin-9 treatment increased TGF-β expression on the surface of THP-1 cells **(** **Figure 5g** **)**. IFN-γ–treated THP-1 cells were able to expand CD4^+^CD25^+^FoxP3^+^CD127^low^ Tregs from two normal patients (**Figure 5h**).

### Galectin-9 Induces Apoptosis of HCV-Specific CTLs

Binding of galectin-9 to Tim-3 has been shown to induce apoptosis of Th1 and alloreactive CD8^+^ T cells [32], leading to attenuation of autoimmune disorders and prolongation of allograft survival [5].

**Figure 6:**
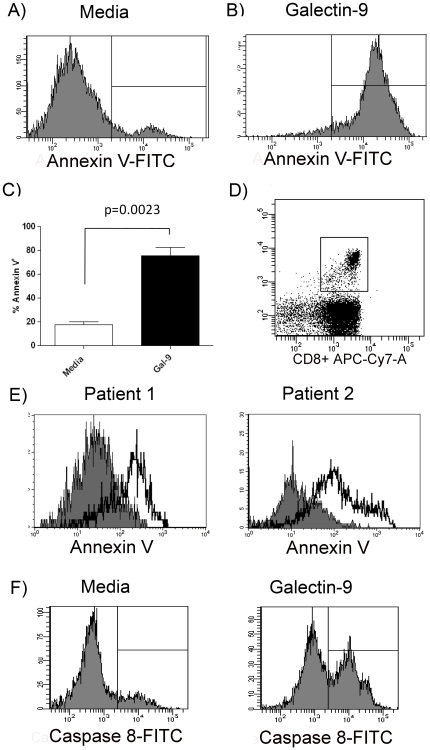
Galectin-9 induces apoptosis of HCV-specific CTLs through caspase-8 activation. CD8^+^ T cells clones specific for NS3∶1406, NS3∶1436 and NS5∶2594 were incubated for 6 hours in media alone (**A**) or with 5 ug/ml of galectin-9 (**B**) followed by staining with Annexin V and 7-AAD. Representative histograms showing the percent of HCV-specific T cells staining with Annexin V following the indicated treatment. (**C**) Combined data for 5 CTL clones from different patients demonstrated an increase in Annexin V/7-AAD with galectin-9 treatment. P-values were calculated using the Wilcoxan matched-pairs signed rank test. (**D**) Plot showing NS3∶1436-specific CD8^+^ T cells by pentamer staining ex vivo. (**E**) Annexin V staining of NS3∶1436- specific CD8^+^ T cells from two HCV patients cultured for 6 hours with media (grey shading) or galectin-9 (solid line, no shading). (**F**). Galectin-9 induces T cell apoptosis through caspase-8 activation. Shown are PBMC from a HCV-positive patient treated for 1 hour with 5 ug/ml galectin-9 or media alone. A FITC-conjugated caspase-8 inhibitor that binds specifically to activated caspase-8 was added for the last hour of culture. Cells were then stained with anti-CD8 and 1436-pentamer and the percentage of HCV-specific T cells with activated caspase-8 was determined by flow cytometric analysis.

Chronic HCV infection is characterized by viral-specific CTLs that demonstrate significant deficits in cytokine production and proliferation, as well as high susceptibility to spontaneous apoptosis and very high expression of PD-1 [33]. We cultured HCV-specific CTL clones with galectin-9 and found that the level of annexin V positivity increased significantly at 6 hrs (**Figure 6a–6c**), and these results were similar in whole PBMCs containing high frequency HLA class I pentamer-positive, HCV-specific T cells (**Figure 6e**). Activation-induced cell death (AICD) is triggered by persistent antigen stimulation and death receptor signaling and is mediated by activation of caspase-8 [34]. We found that 1 hour of galectin-9 treatment activates caspase-8 to induce AICD of HCV-specific CTLs (**Figure 6f**).

## Discussion

Galectin-9 was first described as a T cell–derived factor with eosinophil-specific chemotactic activity, and subsequent work has demonstrated that it plays important roles in the control of effector cells during various phases of the immune response in mammals [35]. On one hand, galectin-9 is a proinflammatory factor that promotes tissue inflammation [36], induces maturation of monocyte-derived dendritic cells [37], and through this process, enhances Th1 immune responses [35]. On the other hand, galectin-9 has a major role in limiting the immune response [38]. Interferon-γ released by Th1/Tc1 cells induces various cell types to produce galectin-9 that, in turn, creates a negative feedback by triggering apoptosis of mature T cells through engagement and stimulation of their Tim-3 receptor [7]. Our findings that galectin-9 triggers pro- but not anti-inflammatory cytokines from hepatic mononuclear cells implicates galectin-9 as a key regulator in inflammatory pathways within the liver that lead to injury. In many models of hepatic 087pathogenesis, levels of TNF-α, the so-called “first” cytokine upon LPS activation of Kupffer cells, are elevated and correlate with extent of injury [18]. Production of proinflammatory cytokines such as TNF-α by immune cells may initially contribute to the control of invading pathogens, including HCV [39]. However, excessive and uncontrolled production of TNF-α may lead to systemic chronic inflammation, induction of hepatic apoptosis, and increased liver fibrosis [40] [41], [42] [43]. We have found galectin-9 to be circulating at significantly higher levels in the serum of HCV-infected patients as compared to normal healthy controls. Patients with non-viral liver disease had intermediate levels of galectin-9 suggesting that it may play an important role in the hepatic immune response. Moreover, we demonstrated that the predominant source of hepatic galectin-9 was Kupffer cells which are liver-resident macrophages. Kupffer cells constitute the first macrophage population with which pathogens, bacterial endotoxins, and microbial debris derived from the gastrointestinal tract come into contact, and together with the sinusoidal endothelial cells, comprise the reticuloendothelial system of the liver [18].

**Figure 7:**
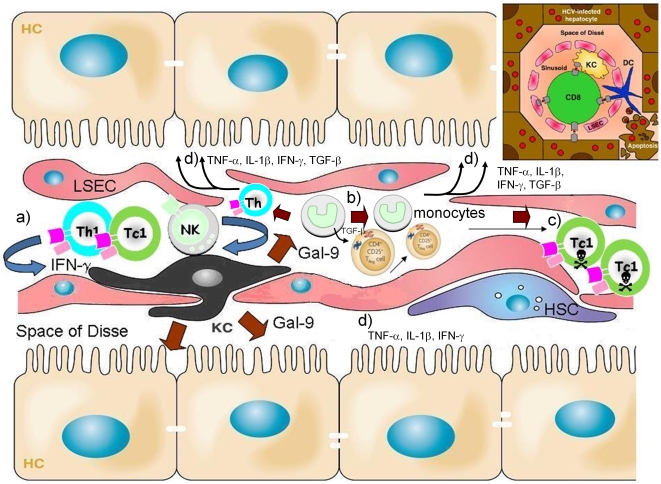
Paradigm for central role of galectin-9 in regulation of hepatitis C immunity. Liver sinusoids are lined by a fenestrated layer of sinusoidal endothelial cells. The unique architecture of the liver allows interaction of antigen-presenting cells with T cells. ***a***) Chronic HCV infection is characterized by hepatic infiltration of Th1, Tc1, NK and NKT cells that secrete IFN-γ which stimulates KCs to produce galectin-9. ***b***) Galectin-9 (large brown arrows) expands CD4^+^CD25^+^FoxP3^+^CD127^low^ regulatory T cells via TGF-β dependent mechanisms. ***c***) HCV-specific CTLs expressing high levels of the Tim-3 receptor are engaged and become apoptotic. ***d***) Galectin-9 also induces production of pro-inflammatory cytokines (TNF-α, IL-1β, IFN-γ) and pro-fibrotic cytokines (TGF-β) that can act on hepatocytes and hepatic stellate cells (HSC). The space of Disse contains the HSC. Hepatocytes (HC), liver sinusoidal endothelial cells (LSEC), Kupffer cells (KC), Th1 (CD4^+^ T cells), Tc1 (CD8^+^ T cells), natural killer (NK) and natural killer T (NKT) cells, hepatic stellate cells (HSC). *Inset*, the strategic location of KCs and the slow blood flow through the sinusoids allows contact with infiltrating lymphocytes (structural relationship of hepatic cells adapted from [18], [45]).

We propose the paradigm shown in **Figure 7**. Previous work has shown that IFN-γ is elevated in chronic HCV infection [42] and associated with progressive liver injury [44]. Immune cells (e.g., activated T cells, natural killer and natural killer T cells, which are particularly enriched in the liver) produce IFN-γ which then stimulates galectin-9 production by hepatic KCs. The strategic location of KCs within hepatic sinusoids [45] allows secreted and cell surface-associated galectin-9 to interact with the Tim-3 receptor on T cells to dampen Th1/Tc1 immunity. KC-derived galectin-9 induces the robust secretion of an array of pro-inflammatory mediators (TNF-α, IL-1-β, IFN-γ) that can further amplify the immunopathology associated with HCV. As a counter-effect, Galectin-9 is found to expand Tregs in a TGF-*β* dependent manner and induce rapid apoptosis of HCV-specific CTLs (recently shown to express the highest levels of Tim-3) [9], attenuating the adaptive effector immune response. Prior work has demonstrated that caspase-1 is involved in galectin-9-mediated apoptosis of the MOLT-4 lymphoblastic leukemia cell line [46]; we found that caspase 8 mediated antigen induced cell death of HCV-specific CTLs.

Secreted galectin-9 has also been implicated in direct Th1 immunosupression by nasopharyngeal cancer cells [35]. We found the highest levels of plasma galectin-9 in HCV-infected patients with HCC suggesting that a similar mechanism may be operating. Secreted galectin-9 from tumor cells or KC may explain the increase in Tregs and decrease in CD8 T cells seen in the peritumor region of HCC [47], [48].

In conclusion, our data provide several new insights into the immunobiology of HCV, offering a possible explanation for the observation that HCV infection is associated with generation and expansion of regulatory T cells, functional impairment and apoptosis of HCV-specific CTLs, and ultimately, the development of viral persistence in the majority of patients. Galectin-9 has pleiotropic roles and may represent a novel therapeutic target in patients with viral or inflammatory diseases of the liver.

## Methods

### Ethics Statement

The study protocol was approved by the Institutional Review Boards at the University of Colorado Health Sciences Center, Denver; and the Oregon Health Sciences University, Portland. Both written and oral consent was obtained before samples were collected.

### Cell Isolation

PBMC and plasma were prepared from whole blood using CPT tubes from Becton Dickinson (Franklin lakes, NJ) per the manufacturer's protocol. PBMCs were also isolated from whole blood using Ficoll (Amersham Biosciences; Piscataway, NJ). PBMCs were viably frozen in 80% fetal bovine serum (BioWhittaker, Walkersville, MD), 10% dimethyl sulfoxide (DMSO), and 10% RPMI 1640 medium (Life Technologies, Grand Island, NY) in liquid nitrogen for subsequent analyses. Plasma was frozen at −80°C until analysis. Hepatic perfusate mononuclear cells were isolated from cadaveric liver transplant donors using centrifugation Ficoll separation as previously described [25], [49]. Hepatic mononuclear cells (HMNCs) were isolated from explanted liver tissue at the time of liver transplantation for HCV-related liver disease. Tissue samples were dissected into 1-mm^3^ pieces and added to complete RPMI 1640 medium and 0.05%collagenase type IV (312 U/mg), and the mixture was incubated at 37°C for 60 min. The supernatant was removed, and cell pellets were diluted in complete RPMI 1640 medium and centrifuged at 125×*g* for 10 min. HMNCs were viably frozen in 80% fetal bovine serum (as described above) for subsequent analysis.

### Galectin-9 ELISA

Using a previously described sandwich ELISA assay [50], [51], we analyzed plasma from 10 normal controls, average age 37; 30% were male. We also analyzed 22 patients with chronic HCV, 7 also had hepatocellular carcinoma (HCC). The average age of these patients was 50 years and 59% were male. In the HCV group that included HCC patients, 67% of patients had cirrhosis (stage IV) clinically or by liver biopsy, 4.5% were stage III, 9.5% were stage II and 19% were stage I.

In brief, 96-well plates (Nunc, Naperville, IL) were coated with an anti-human Galectin-9 MoAb (9S2-3, GalPharma, Japan), blocked with 3% fetal bovine serum containing 0.05% Tween 20 in PBS, then incubated for 1 hour at 37°C with 8-fold-diluted plasma. After several washings, Galectin-9 remaining in the wells was recognized by polyclonal anti-human Galectin-9 antibody conjugated with biotin using EZ-Link Sulfo-NHS-Biotin reagent (Pierce). Quantification was performed using streptavidin-conjugated horseradish peroxidase (Invitrogen, Tokyo, Japan) and the colorimetric substrate tetramethylbenzidine (KPL, Gaithersburg, MD), and the optical density was read with a microplate spectrophotometer (Bio-Rad).

### Galectin-9 Immunohistochemistry

Paraffin-embedded liver biopsy and liver resection specimens were stained for Galectin-9 and CD68. Normal tissue was obtained from patients being evaluated by liver biopsy for live donor transplantation (n = 5) or patients undergoing resection of metastatic adenocarcinoma (n = 10). The average age of these patients was 51, and 53% were male. Thirty nine patients with chronic hepatitis C were analyzed with an average age of 52, 64% were men. Forty eight percent were fibrosis stage I, 36 percent were stage II, 8 percent were stage III and eight percent had stage IV fibrosis (cirrhosis).

The goat polyclonal galectin-9 antibody (R&D Systems; Minneapolis, MN) was used at a dilution of 1∶100 for large tissues and 1∶200 for biopsies. The antibody was diluted in Background Reducing Diluent (Biocare Medical; Concord, CA) to reduce background staining of non-specific proteins. The CD68 antibody (clone KP-1; DAKO, Carpenteria, California) was diluted to 1∶2000 in PBS pH 7.4 + 1% BSA + 0.05% Sodium Azide. Antigen retrieval was performed using pH 9.5 BORG solution (Biocare Medical; Concord, CA) in a pressure cooker for 5 minutes at 125°C, (22 psi). The slides were cooled on the benchtop for 10 minutes after retrieval. All of the staining steps were performed in the Ventana NexES autostainer (Ventana Medical Systems; Tucson, AZ) at 37°C. An I-VIEW DAB detection kit (Ventana) was used for antibody detection and included endogenous peroxidase block, secondary antibody, streptavidin horseradish peroxidase, DAB substrate with hydrogen peroxide activator and copper enhancer. A biotinylated rabbit anti-goat secondary antibody (Jackson ImmunoResearch; West Grove, PA) used at a 1∶50 dilution in PBS, pH 7.6 was used in place of the secondary antibody included in the I-View kit. Following the 16 minute primary antibody incubation, the secondary and SA-HRP antibodies were applied for 8 minutes respectively. Visualization was achieved with DAB plus hydrogen peroxide activator for 5 minutes. The process was completed with the addition of copper sulfate for 4 minutes for final color enhancement. The slides were then counterstained with Mayer's hematoxylin for 2 minutes at room temperature and the nuclei stained blue in 1% ammonium hydroxide in water. The slides were dehydrated, cleared and mounted with synthetic resin for microscopic evaluation.

Staining for galectin-9 and CD68 was analyzed and scored by a single pathologist (M.S.) in a blinded fashion. Intensity was graded on a scale from 0 to 3+ (4 total values) and frequency of staining was graded as 0 (<10%); 1 (10–40%); 2 (40–70%); 3 (>70%).

### Immunofluorescence and Confocal Microscopy

Immunostaining of 5 µm sections from paraffin-embeded liver biopsies was performed using a combination of the following markers, Galectin-9 (goat-anti-human Galectin-9, 1∶100, R&D Systems), CD-68 or Albumin (mouse-IgG-anti CD68 or mouse-IgG-anti Albumin, 1∶100, Abcam) in combination with DAPI for nuclear staining (1∶20,000, Invitrogen). Sections were cleared in xylene and blocked for 1 h with 10% Normal Goat Serum and 1% bovine serum albumin in phosphate-buffered saline (block solution) and then incubated over- night at 4°C with primary antibody diluted in 10% block solution. Sections were washed with phosphate-buffered saline and stained with the appropriate secondary antibodies: rabbit anti-goat Alexa 546 and donkey anti-mouse Alexa 488 (1∶500; Invitrogen). Sections were mounted using Mowiol 4–88 (Calbiochem) containing 2,5% of 1,4-diazabicyclo[2.2.2]octane (DABCO, Sigma). Immunoreactivity was not detected in the absence of primary antibodies. Images were captured using a Zeiss LSM 510 META system on Axiovert 200 M microscope with Zeiss plan-apochromat 40x/1.2 objective. Images were assembled in Adobe Photoshop.

### Monocyte Purification, Cell Culture and Western Blotting

CD14^+^ cells were isolated by positive selection using magnetic beads (Miltenyi Biotech; Auburn, CA) according to the manufacturers protocol. CD14^+^ cells were greater than 95% pure. CD14^+^ cells were cultured at 1×10^6^/ml in 25 ng/ml MCSF (R&D Systems; Minneapolis, MN) for 48 hours in RPMI (Invitrogen; Carlsbad, CA) + 10% fetal bovine serum in to differentiate them into macrophages. IFN-γ, IL-1β, and LPS were from R&D Systems. Core protein and the β-gal control were from Virogen (Boston, Massachusetts). Galectin-9 protein was purified and confirmed to be LPS free as described previously. [37]

Cells were stimulated for 48 hours and cell lysates were collected in 5XSDS-PAGE sample buffer (Sigma-Aldrich, St. Louis, MO). Protein was quantitated using a BCA Assay Kit (Pierce; Rockford, IL) and 10 ug per lane was run on a 10% SDS-PAGE gel. Western blotting was performed using BioRad (Hercules, CA) mini gel protocol and reagents. Protein was transferred to PVDF membranes (Bio-Rad; Hercules, CA). Chemiluminescent reagents (Pierce; Rockford, IL) and the following antibodies were used for detection; a polyclonal goat anti-human galectin-9 antibody (R&D Systems) used at 1∶1000, followed by a donkey anti-goat HRP secondary (R&D Systems). Densitometry analysis was performed using Image J software from the NIH.

### Galectin-9 Real Time PCR

THP-1 cells were cultured at 10^6^/ml with and without Interferon-γ (20 ng/ml) for the times indicated. RNA was isolated using Trizol (Invitrogen; Carlsbad, CA) and 1 µg RNA was used for reverse transcription. Validated primers and probes for gal-9 TaqMan RT-PCR were obtained from Applied Biosystems (Foster City, CA) Gene Expression Assays (LGALS9, #HS_00247135) and used according to the manufacturer's recommendations with 1/20^th^ of the RT reaction [36]. GAPDH was used as the reference control and relative expression was calculated using the ΔΔCt method.

### Induction of Cytokines by Galectin-9

Hepatic or peripheral mononuclear cells (2×10^6^/ml) were cultured in 96 well plates (200 ul/well) for 48 hours in media alone (RPMI+10% human serum) ± Galectin-9 (5 ug/ml). After the incubation period, supernatant was collected and frozen for subsequent cytokine analysis. Thawed supernatants were transferred to MultiScreen filter plates (Millipore; Billerica, MA) and assayed using the human cytokine/chemokine Milliplex™ MAP Kit 96-well plate assay (Millipore) using a Luminex^100^ IS System (Luminex Corp; Austin, TX) to determine the quantities of pro (IFN-γ, IL-1β, TNF-α) and anti-inflammatory (IL-4, IL-10 and IL-13) cytokines. Duplicate samples and standards were processed according to the manufacturer's protocol. Results were analyzed using 4-parameter logistic curves (fluorescence intensity vs. pg/ml) generated by Luminex^100^ IS Software (versions 2.2 and 2.3).

### Treg Induction and Analyses

Peripheral mononuclear cells (2×10^6^/ml) or CD14-depleted mononuclear cells were cultured for 5 days in media alone (RPMI+10% human serum) ± Galectin-9 (5 ug/ml). CD14^+^ cells were depleted using anti-CD14 magnetic isolation beads (Miltenyi Biotech; Auburn, CA). For some cultures anti-TGF-β antibody (anti-human LAP, 10 ug/ml) (R&D Systems; Minneapolis, MN) was added at day 0 and day 2 of culture. Co-culture experiments using the THP-1 monocyte cell line (ATCC; Manassas, VA) ± IFN-γ (20 ng/ml for 48 hours) and bead-purified CD4^+^ T cells (Miltenyi Biotech), >90% purity) were carried out at a 1∶1 ratio for 5 days. Treg levels were assessed by flow cytometic analysis. Multi-color multiparameter flow cytometry was performed using a FACSCanto II instrument (BD Biosciences) compensated with single fluorochromes and analyzed using Diva™ software (BD Biosciences). Fluorochrome-labeled monoclonal antibodies (MAb) specific for CD3, CD4, CD8 and CD25 were obtained from BD Biosciences (San Jose, CA). Anti-CD127 was supplied by R&D Systems (Minneapolis, MN). Cryopreserved PBMCs (1–2×10^6^) were stained for cell surface antigen expression by incubating with antibody at 4°C for 30 minutes in the dark. Cells were, washed twice in 2 ml phosphate-buffered saline (PBS) containing 1% bovine serum albumin and 0.01% sodium azide (FACS Wash). Intracellular staining for FoxP3 was carried out using the APC anti-human FoxP3 staining set (eBioscience; San Diego, CA) according to the manufacturer's instructions. Fluorescence minus one (FMO) controls were used to determine background levels of staining.

### Quantification of TGF-β Transcripts in Monocytes

Monocytes were isolated from peripheral blood mononuclear cells using anti-CD14 magnetic beads (Miltenyi Biotech), >90% purity). CD14^+^ monocytes (2×10^6^/ml) were cultured in 96-well plates (200 ul/well) for 48 hours in media alone (RPMI+10% human serum) ± Galectin-9 (1 ug/ml). After the culture period, cell pellets were harvested and frozen for subsequent PCR analysis. RNA was isolated using the RNeasy Mini Kit and converted to cDNA using the QuantiTect RT kit (both from Qiagen, Valencia, CA, standard protocols). Real Time Quantitative PCR was carried out on a 7300 Real Time PCR system (Applied Biosystems, Carlsbad, CA). The TGF-β primer set was purchased from Qiagen (Hs_TGFB1_1_SG, QT00000728). Samples were run in duplicate in a 25 ul reaction volume consisting of 12.5 ul of SYBR Green PCR Mix (Qiagen), 2.5 ul of the primer set, 2.5 ul of cDNA and 7 ul of H_2_O. Cycling conditions consisted of 40 cycles (94° for 15 seconds, 50° for 30 seconds, 72° for 30 seconds). Each individual sample was normalized to β-actin and TGF-β in Galectin-9 treated samples was compared to the matched media alone sample. Fold change in TGF-β transcripts was estimated using the ΔΔCT method.

### T Cell Cloning

PBMCs from HCV-positive patients were stained with anti-CD8 and HLA-A2 Pro5 MHC pentamers with the NS3∶1406–1415 or NS5∶2594–2603 peptide or an HLA-A1 pentamer with the NS3∶1426–1444 (ProImmune; Springfield, VA). Pentamer positive cells were single cell sorted into 96-well round bottom plates containing 1×10^5^ irradiated autologous feeder cells and 5×10^3^ LCL using a FACSAria multi-color high-speed sorter (BD Biosciences). Wells were stimulated with 0.05 ng/ml anti-CD3 antibody (eBioscience) and 3 ng/ml IL-2 and wells were stained with pentamer after 3–5 weeks. HCV-specific T cell clones were maintained by stimulating with anti-CD3 and IL-2 every 14 days in RPMI + 10% human serum (Gemini Bio-Products, Sacramento, CA).

### T Cell Apoptosis Assay

PBMCs from chronic HCV patients or HCV-specific T cell clones were stimulated with 5 ug/ml recombinant galectin-9 for 6 hours at 37°C. Cells were stained with fluorochrome-labeled monoclonal antibodies (MAb) specific for human CD3- Pacific Blue, CD4- APC, CD8-PerCp (BD Biosciences) and Pro5 MHC pentamer for 1 hour at 4°C in the dark. Cells were washed twice with 2 ml FACS Wash and subsequently resuspended in 1X Annexin V Binding Buffer (BD Biosciences) at a concentration of 1×10^6^ cells/ml. Cells were stained with Annexin V-FITC and the vital dye 7-AAD for 15 minutes at room temperature in the dark and analyzed by Multiparameter flow cytometry using a BD FACSCanto II instrument (BD Biosciences). For analysis of active caspase 8 and caspase 9 cells were stimulated for 1 hour with 5 ug/ml galectin 9 and incubated with FITC-IETD-FMK (caspase 8) or RED-LEHD-FMK (Caspase 9) according to the caspase detection kit protocol (Calbiochem).

### Detection of TGF-β on the Surface of THP-1 Cells

THP-1 cells were cultured +/− Galectin-9 (2.5 ug/ml) for various times (overnight to 72 hours). For the detection of active TGF-β1, cultured THP-1 cells were incubated with and without LAP (10 ug/ml) for two hours at 4°C followed by staining for anti-LAP-PE (TGF-β1) or isotype matched control (R&D Systems) [52].

## Supporting Information

Figure S1 Galectin-9 Immunofluorescence in HCV liver. Paraffin embedded liver biopsy specimens were stained with antibodies to the proteins indicated and analyzed by confocal microscopy. A. Galectin-9/Albumin/DAPI staining. B. Galectin-9/CD68/DAPI staining. Double positive staining (yellow) indicated by white arrows. The white bar denotes 10 µm.
(1.99 MB TIF)

Figure S2 Interferon-γ induces Galectin-9 mRNA in THP-1 cells. 10^6^ THP-1 cells/ml were cultured in 20 ng/ml IFN-γfor the times indicated in triplicate. RNA was isolated, reverse transcribed and analyzed by TaqMan real time PCR using GAPDH as a control. P-values were calculated using a t-test.
(4.24 MB TIF)
