# Correction: A Crucial Role for Kupffer Cell-Derived Galectin-9 in Regulation of T Cell Immunity in Hepatitis C Infection

**DOI:** 10.1371/annotation/d15b793c-85c7-4529-bc80-aabcb088a8cf

**Published:** 2010-03-12

**Authors:** John A. Mengshol, Lucy Golden-Mason, Tomohiro Arikawa, Maxwell Smith, Toshiro Niki, Ryan McWilliams, Jessica A. Randall, Rachel McMahan, Michael A. Zimmerman, Manu Rangachari, Evgenia Dobrinskikh, Pierre Busson, Stephen J. Polyak, Mitsuomi Hirashima, Hugo R. Rosen

The first and second authors of this article, John A. Mengshol and Lucy Golden-Mason, should be listed as having contributed equally to this work.

