# Supplementary figures and images for: A Crucial Role for Kupffer Cell-Derived Galectin-9 in Regulation of T Cell Immunity in Hepatitis C Infection
Source: PLoS One. 2010 Mar 4;5(3):e9504. doi: 10.1371/journal.pone.0009504 (PMC2831996; doi:10.1371/journal.pone.0009504)

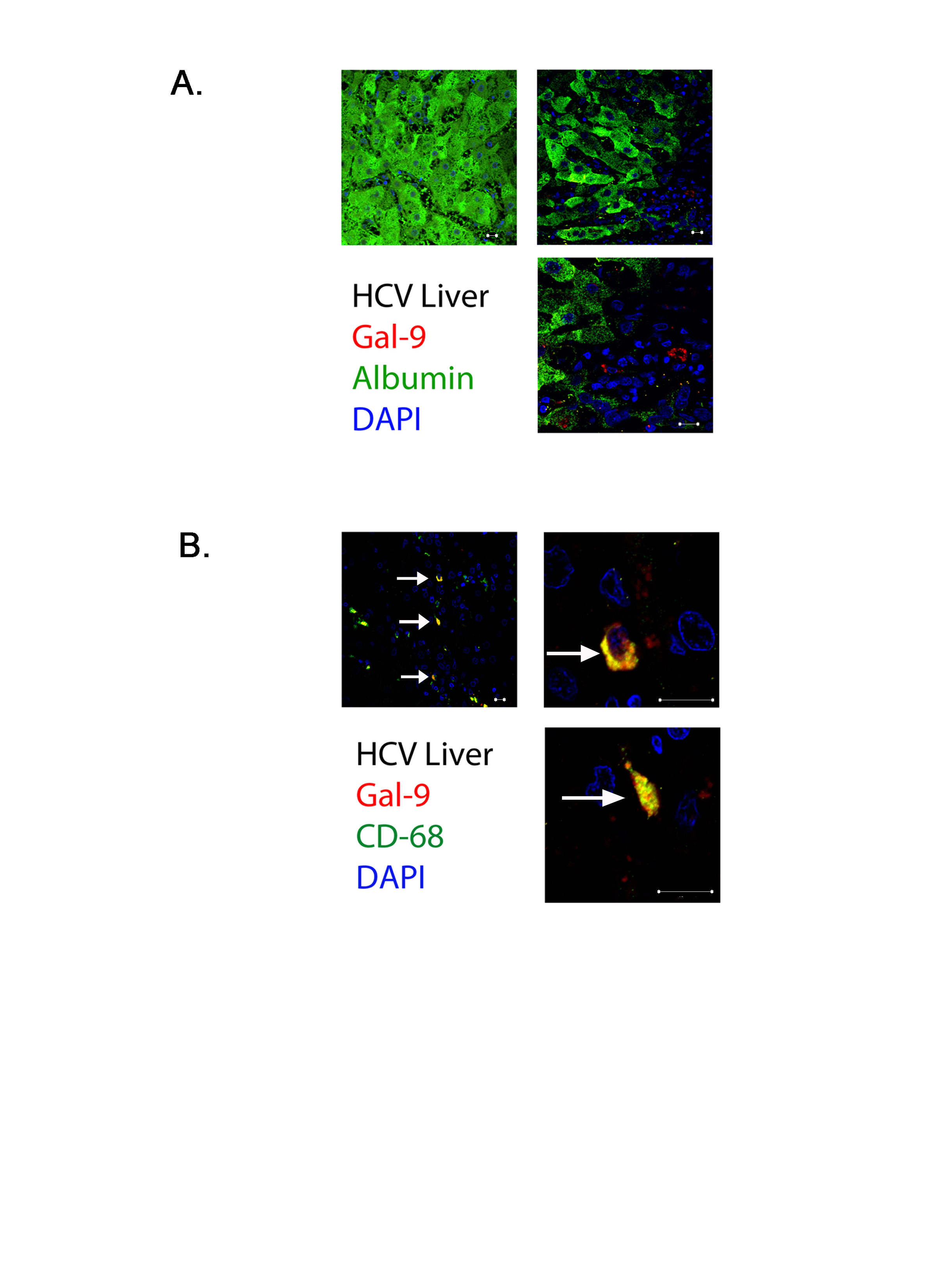

Supplement: Figure S1 — Galectin-9 Immunofluorescence in HCV liver. Paraffin embedded liver biopsy specimens were stained with antibodies to the proteins indicated and analyzed by confocal microscopy. A. Galectin-9/Albumin/DAPI staining. B. Galectin-9/CD68/DAPI staining. Double positive staining (yellow) indicated by white arrows. The white bar denotes 10 µm. (1.99 MB TIF) [file pone.0009504.s001.tif]
